# Supplementary material for: Synergistic Activity of Cefiderocol in Combination with Avibactam, Sulbactam or Tazobactam against Carbapenem-Resistant Gram-Negative Bacteria
Source: Cells. 2024 Aug 6;13(16):1315. doi: 10.3390/cells13161315 (PMC11352988; doi:10.3390/cells13161315)
Supplement: Supplementary file 1 [file cells-13-01315-s001.zip › cells-3071082-supplementary.pdf]

Table S1. Phenotypic characteristics of CR strains included in this study. Reduced susceptibility to antimicrobial antimicrobials is indicated in bold.

**Isolate**	**MIC (mg/L)[1]**															
	**AZT[2]**	**C/T[3]**	**COL[4]**	**P/T[5]**	**TGC[6]**	**IMI[7]**	**IMR[8]**	**FEP[9]**	**CZA[10]**	**AMI[11]**	**ERV[12]**	**CFD[13]**	**FOS[14]**	**MERO[15]**	**TOB[16]**	**MEV[17]**
**CRE CFD-susceptible**	**>32**	**>8/4**	≤0.5	**>32/4**	≤0.5	**>8**	0.5/4	**>16**	1/4	≤2	0.25	0.5	≤16	**>16**	≤0.5	0.12/8
**CRE CFD-resistant**	**>32**	**>8/4**	**>16**	**>32/4**	**1**	**>8**	**>8/4**	**>16**	**>14/4**	**16**	**>0.5**	**>8**	32	**>16**	**>4**	**16/8**
**CR-Pa CFD-susceptible**	**>32**	**>8/4**	**>16**	8/4	**>1**	**>8**	**8/4**	**16**	4/4	8	**>0.5**	2	**>64**	**>16**	**>4**	**>16/8**
**CR-Pa CFD-resistant**	**16**	**>8/4**	1	**16/4**	**>1**	**>8**	2/4	**>16**	**16/4**	32	0.5	**4**	**>64**	4	**>4**	4/8
**CR-Ab CFD-susceptible**	**32**	**>8/4**	**8**	**>32/4**	≤0.5	**>8**	**>8/4**	**>16**	**>16/4**	>32	0.25	0.5	**64**	**>16**	**>4**	**>16/8**
**CR-Ab CFD-resistant**	**>32**	**>8/4**	≤0.5	**>32/4**	≤0.5	**>8**	**>8/4**	**>16**	**>16/4**	>32	0.12	**>8**	32	**>16**	**>4**	**>16/8**

[1] Applying EUCAST breakpoint; [2] AZT, aztreonam; [3] C/T, ceftolozane/tazobactam (constant 4 mg/L); [4] COL, colistin; [5] P/T, piperacillin/tazobactam (constant 4 mg/L); [6] TGC, tigecycline; [7] IMI, imipenem; [8] IMR, imipenem/relebactam (constant 4 mg/L); [9] FEP, cefepime; [10] CZA, ceftazidime/avibactam (constant 4 mg/L); [11] AMI, amikacin; [12] ERV, eravacycline; [13] CFD, cefiderocol; [14] FOS, fosfomycin; [15] MERO, meropenem; [16] TOB, tobramycin; [17] MEV, meropenem/vaborbactam (constant 8 mg/L).
